# Single Nucleotide Polymorphism of *TYK2* Gene and Susceptibility to Rheumatoid Arthritis in Iranian Population

**Published:** 2019

**Authors:** Azadeh Mohamadhosseini, Reza Mansouri, Ali Javinani, Amir Ashraf Ganjouei, Massoumeh Akhlaghi, Saeed Aslani, Elham Hamzeh, Ahmadreza Jamshidi, Nooshin Ahmadzadeh, Mahdi Mahmoudi

**Affiliations:** 1. Department of Immunology, Faculty of Medicine, Shahid Sadoughi University of Medical Sciences, Yazd, Iran; 2. Rheumatology Research Center, Tehran University of Medical Sciences, Tehran, Iran

**Keywords:** Rheumatoid arthritis, Single nucleotide polymorphism, TYK2 kinase

## Abstract

**Background:**

Rheumatoid Arthritis (RA) is a debilitating disorder in which the immune system mainly targets the synovial tissue. Janus kinase family including tyrosine kinase 2 (TYK2) is one of the crucial mediators of the downstream signaling pathway of inflammatory cytokines that further contributes to RA pathogenesis. In this study, the association of *TYK2* gene rs34536443 polymorphism, which may affect the function of TYK protein and, hence, the inflammatory settings, with RA susceptibility was investigated. Moreover, its correlation with demographic and serological features of the patients was assessed.

**Methods:**

In the present study, 700 RA patients and 700 sex, age and ethnicity-matched healthy individuals as the control group were included. MGB TaqMan real-time allelic discrimination method was used to determine the rs34536443 polymorphism. Rheumatoid factor, anti-cyclic citrullinated peptide antibody, erythrocyte sedimentation rate and C-reactive protein were also measured.

**Results:**

The frequency of rs34536443 minor allele (C allele) was not different between patients and control group [1.7 *vs.* 2.61 percent, OR (95% CI)=1.35 (0.78–2.33);p=0.27]. There was not a statistically significant association between rs34536443 genotypes and RA susceptibility. Genotypes of rs34536443 polymorphism were associated nor with demographic neither with serological features of RA patients.

**Conclusion:**

In the present study, there was not any association between *TYK2* gene rs34536443 polymorphism with either disease susceptibility, demographic and serological features of Iranian RA patients. These findings are not compatible with previous works from other ethnicities, further supporting the role of genetics in disease susceptibility.

## Introduction

Rheumatoid Arthritis (RA) is an auto-immune disease characterized by inflammatory cascades primarily happening in joints but the other organs, albeit less so, can be affected 1. According to the World Health Organization (WHO) regions classification, the prevalence of RA was reported in a range from 0.37% in Eastern Mediterranean region to 1.25% in American region 2. RA exhibits a strong gender predilection with the female to male ratio of 4.7:1 in low and middle-income countries 2. RA has the estimated disability-adjusted life years of 4.8 million in 2010 which was ranked as the 42^nd^ contributor to the global disability 3. Thus, according to its relatively high prevalence andmorbidity, RA is one of the major fields of interests in Rheumatology and Immunology.

In RA, arthritis-associated antigens are presented by dendritic cells to naïve CD4+ T cells 4. Following their activation and differentiation toward the T Helper (TH) 17 cells, the immune system orchestrates an inflammatory cascade with a high spectrum of cytokines production. These cytokines affect a wide range of receptors, which are linked to the signaling mediators from Janus Kinase (JAK) family 5. JAK family has four major members; JAK1, JAK2, JAK3, and tyrosine kinase 2 (TYK2) 6. They are non-receptor tyrosine kinases, which are activated after the binding of cytokines to their relative receptors. Following their activation, they make a docking site for signaling molecules, mostly members of the Signal Transducer and Activator of Transcription (STAT) family 6. STAT molecules make a dimer after phosphorylation which then enter the nucleus and induce the expression of further molecules of the inflammatory cascade 5.

TYK2 is linked to the cytoplasmic domain of type I and II cytokine receptors, which are involved in the signaling pathway of interleukin (IL)-6, IL-12 and IL-23 5. Its gene is located in 19p13.2 position andits polymorphismshave been linked to a variety of auto-immune diseases 7. It has been reported previously that *TYK2* gene polymorphisms influenced the susceptibility to RA 8, Systemic Lupus Erythematosus (SLE) 9, systemic sclerosis 10, Inflammatory Bowel Disease (IBD) 11 and Multiple Sclerosis (MS) 12. The present study aimed to investigate the rs34536443 Single Nucleotide Polymorphism (SNP) of *TYK2* gene in Iranian RA patients. Also, its association with demographic and serological features of the patients was described as well.

## Materials and Methods

### Study population

This study was conducted on 700 RA patients from the outpatient’s rheumatology clinic of Shariati Hospital who were diagnosed based on the American College of Rheumatology (ACR)/European League Against Rheumatism (EULAR) 2010 RA classification criteria 13. Control group of this study composed of 700 age, sex, and ethnicity matched healthy individuals. Written informed consent was obtained from all the participants. The present study was approved by the ethics committee of Yazd University of Medical Sciences.

### DNA preparation and polymorphism analysis

For all participants, peripheral blood was collected into tubes with EDTA as an anticoagulant and Erythrocyte Sedimentation Rate (ESR) was measured. Genomic DNA was extracted using standard proteinase K digestion- phenol/chloroform extraction method 14. The extracted DNA samples were stored at −20*°C*. Approximately, 20 *ng* of the genomic DNA of each sample was used for genotyping. Genotyping of *TYK2* gene rs34536443 was performed using the MGB-TaqMan Allelic Discrimination method (Applied Biosystems, Foster City, CA, USA). Amplification was performed in 10 *μl* reaction volumes, containing 5 *μl* of the TaqMan Genotyping master mix, 0.25 *μl* of TaqMan Genotyping assay mix, 0.25 *μl* of distilled water, and 4.5 *μl* of genomic DNA.

### Serologic analysis

The plasma samples were obtained after centrifuging the patients whole blood samples, which were drained previously into EDTA-containing test tubes. The Rheumatoid Factor (RF) and anti-Cyclic CitrullinatedPeptide antibody (anti-CCP), and C-Reactive Protein (CRP) were measured by commercially available kits.

### Statistical analysis

Control group was tested to be in the Hardy-Weinberg Equilibrium. The frequency of alleles and genotypes were compared between RA patients and control group using chi-square test. The mean comparison of ESR level, RF and anti-CCP titers of different genotypes in case group was done by ANOVA. p-values less than 0.05 were considered statistically significant. All analyses were performed using SPSS package for Windows (Version 19.0, IBM SPSS Inc., USA).

## Results

The demographic and clinical characteristics of our study population are shown in table 1. The mean age of the RA patients was 50.32±11.27 years whereas the mean age of control group was 48.44±12.37 years. The female to male ratio was 3.48:1 in patients group and 6.52:1 in healthy individuals. The mean ESR level was higher in patients (20.64±17.14 *vs*. 10.27±7.95), but the healthy individuals had a higher percentage of positive CRP (61.1 *vs*. 46%). The means of RF and anti-CCP titer were 85.5±7.63 and 138.7±18.96 international unit (*IU*)/*ml*, respectively among RA patients.

**Table 1.** Demographic and clinical data of RA patients and healthy controls

**Characteristics**	**RA patients (n=700) n (%)**	**Healthy controls (n=700) n (%)**
**Gender**		
Male	156 (22.2%)	93 (13.3%)
Female	544 (77.8%)	607 (86.4)
**Smoking**		
Positive	-	35 (5%)
Negative	-	665 (95%)
**CRP**		
Positive	322 (46%)	428 (61.1%)
Negative	378 (54%)	272 (38.9%)
**ESR**	20.64 ±17.14	10.27±7.95
**Age (yr)**	50.32 ± 11.27	48.44 ±12.37
**RF**	85.5 ± 7.63	-
**Anti-CCP**	138.7±18.96	-

The allele and genotype frequencies of rs34536443 in RA patients and control group are shown in table 2. The frequency of minor allele (C allele) was not different among patients and healthy individuals (1.7 *vs*. 2.61%, OR (95% CI)=1.35 (0.78–2.33), p-value= 0.27). The frequencies of CC and GC genotypes were negligible; however, according to our analyses, there was not a statistically significant difference between the genotypic distribution of the case and control groups. Moreover, the dominant model of CC+CG had no significant differences between patients and controls. Finally, the association of genotype frequencies with clinical characteristics of RA patients is shown in table 3. There was not any correlation between genotype frequencies and patients' age and gender, ESR level, and the titer of RF and anti-CCP.

**Table 2.** Allele and genotype distribution of *TYK2* gene rs34536443 SNP in RA patients and healthy controls

**SNP**	**Allele/Genotype**	**RA(n=700) n (%)**	**Control (n=700) n (%)**	**OR (95% CI)**	**p-value**
**rs34536443**					
	C	31 (1.70%)	23 (2.61%)	1.35 (0.78–2.33)	0.27
	G	1369 (98.30%)	1377 (97.39%)	**Reference**	
	CC	1 (0.14%)	0 (0%)	3 (0.12–73.98)	0.50
	CG	8 (1.14%)	7 (1%)	0.14 (0.41–3.17)	0.79
	CC+CG	9 (1.28%)	7 (1%)	1.28 (0.47–3.48)	0.61
	GG	691 (98.71%)	693 (99%)	**Reference**	
HWE			p= 0.05		

RA: Rheumatoid Arthritis; HWE: Hardy–Weinberg Equilibrium

**Table 3.** Association of genotypes of *TYK2* gene rs34536443 SNP with characteristics of the RA patients

**Clinical characteristics**	CC*	**CG**	**GG**	**p-value**
**Age**	30 ^*^	54.25±13.27 ^ $ ^	50.27±11.15	0.12
**ESR**	10	27.62±34.55	20.43±16.28	0.42
**RF**	15.9	44.55±24.96	85.50 ±7.63	0.43
**Anti-CCP**	17	58.28±32.02	142.24±19.67	0.64
**Gender**				
Male	0 (0%)	6 (0.8%)	149 (21.3%)	0.785
Female	3 (0.4%)	19 (2.7%)	523 (74.7%)	

* The data was available for only one patient with CC genotype for *TYK* 2 gene rs34536443 SNP.

## Discussion

In the present study, an attempt was made to investigate the association of *TYK2* gene rs34536443 polymorphism with RA susceptibility. According to our results in Iranian population, rs34536443 polymorphisms do not have any influence on RA development. Furthermore, neither demographic features nor RF and the anti-CCP titer was correlated with rs34536443 polymorphisms.

The importance of *TYK2* gene polymorphisms in autoimmune diseases was supported by a vast majority of the studies. The previously studied works on *TYK2* gene polymorphisms in RA are shown briefly in table 4. According to a meta-analysis published in 2011, the rs34536443 and rs2304256 SNPs were associated with auto-immune inflammatory disorders including RA, SLE, MS and IBD 15. But, at that time, there was not any published evidence on rs34536443 polymorphism and RA susceptibility. In 2012, the Genome-Wide Association Study (GWAS) on European RA patients, have reported the association of rs34536443 with RA for the first time 8. According to results from the Immunochip array, G allele of rs34536443 had a protective effect on RA development. After regrouping the patients based on anti-CCP status, the association remained significant only in seropositive individuals. These findings were also replicated for combined analysis of Immunochip results and imputed GWAS data from non-overlapping samples. In line with mentioned discoveries, Diogo *et al* showed that the three protein-coding variants in *TYK2* gene, including P1104A (rs-34536443) have a protective effect against RA in European patients 16. Additionally, they showed that the same three variants also protect against SLE and IBD. Moreover, based on the phenome-wide association study, they reported that the P1104A was not associated with non-autoimmune phenotypes. Their findings can support further the role of TYK2 targeting as the possible promising treatment for these auto-immune diseases.

**Table 4.** Association of *TYK2* gene polymorphism with RA susceptibility in different populations

**Ethnicity**	**Number of cases**	**Allele**	**Odds ratio**	**p-value**	**Reference**
**European**	11,475	G	0.62	2.3×10− ^ 14 ^	(8)
**European (US, UK, Germany)**	2816	G	0.56 (0.47–0.67)	1×10− ^ 10 ^	(17) (JIA)
**Greek**	125	C	2.45 (0.53–11.55)	0.34	(18) (JIA)
**Greek**	392	C	0.60 (0.27–1.35)	0.25	(19)
**Iranian**	700	C	1.35 (0.78–2.33)	0.27	Present study

JIA: Juvenile Inflammatory Arthritis

According to the analyses of the Immunochip array on 2816 European patients with Juvenile Idiopathic Arthritis (JIA), it was showen that G allele of rs-34536443 had a protective effect against oligoarticular and RF-negative polyarticular JIA 17. In contrast to this study, no association was found between *TYK2* gene rs34536443 SNPs and JIA development either at an allelic or genotypic level among Greek patients 18. Unexpectedly, in a study performed on Greece adult RA patients, Myrthianou *et al* revealed that the GC genotype of rs34536443 SNP had a protective role against RA with an OR of 0.33 (0.123–0.90) 19.

In a functional point of view, it has been shown previously that the rs34536443 SNP, located in protein-coding region of *TYK2* gene (Assembly: GRCh37. p13, annotation release: 105, Chromosome: 19, Location: NC_000019.9), can influence the three-dimensional structure of TYK2, which can consequently affect its function 18. Moreover, studies on MS patients revealed that the protective C allele of rs34536443 SNP mediates the divergence of T lymphocytes differentiation toward a TH2 phenotype 20. They showed that the downstream pathways of TYK2, which were activated by interferon (IFN)-β, IL-6 and IL-10were less pronounced in carriers of GC genotype compared to GG carriers. In addition, they reported that the *GATA3* expression was higher in GC genotype carriers, which can favor the TH2 modulation pathway. Besides, the secretion level of IL-5 and IL-13 was also higher in GC carrier T cells upon activation by CD3/CD28 signaling. Nonetheless, no correlation between rs34536443 polymorphisms and M1/M2 balance of macrophages phenotype was identified in their work. Although these data are governed by MS, according to the crucial role of TH1 and TH17 in RA pathophysiology 21, this scenario might also be implicated in RA. Confusingly, a certain piece of evidence described that despite the catalytic impairment of TYK2 P1104A, it has a comparable IFN-α signaling potency relative to the wild-type protein 22.

According to the intimate contribution of JAK family proteins in inflammatory pathways, there was a great interest to target them for the treatment of inflammatory disorders. Tofacitinib, a monoclonal antibody, is a JAK inhibitor approved for RA treatment with long-term safety and efficacy 23. Nevertheless, Tofacitinib targets more specifically the JAK3 and JAK1 with less affinity for TYK2 24. Moreover, it was described that the Tofacitinib could bind to the adenosine triphosphate-binding cavity of TYK2, which can further diminish its potential to activate the STAT molecules 25. Recent studies have indicated promising outcomes about the efficacy of TYK2 inhibitors in SLE and IBD treatment *via* blocking the IL-12, IL-23, and IFN-I pathways in animal models with short-term safety profile in human 26,27.

## Conclusion

In general, JAK family has a key role in mediating the inflammatory responses in auto-immune diseases. TYK2 is one of the family members that its gene polymorphisms were associated with RA susceptibility in different populations (Table 4). However, in the present work, no association was found between the rs-34536443 polymorphism and RA. These findings can further support the determinative role of ethnicity in the etiopathogenesis of such diseases.
